# Multiplexing Proteomic and Ingenuity Pathway Analysis of Attention/Working Memory in Virally Suppressed Women with HIV: A Feasibility Study

**DOI:** 10.3390/diagnostics15202649

**Published:** 2025-10-21

**Authors:** Wei Li, Leah H. Rubin, Yanxun Xu, Yuezhe Wang, Raha Dastgheyb, Travis Ptacek, Ge Wang, Mirjam-Colette Kempf, Jodie A. Dionne, Deborah Konkle-Parker, Daniel Y. Li, Anandi Sheth, Igho Ofotokun, David E. Vance

**Affiliations:** 1 School of Health Professions, University of Alabama at Birmingham, Birmingham, AL 35233, USA; 2 Bloomberg School of Public Health, Johns Hopkins University, Baltimore, MD 21205, USA; 3 Whiting School of Engineering, Johns Hopkins University, Baltimore, MD 21218, USAyuezhewang@gmail.com (Y.W.); 4 School of Medicine, Johns Hopkins University, Baltimore, MD 21287, USA; 5 CCTS Bioinformatics, School of Medicine, University of Alabama at Birmingham, Birmingham, AL 35233, USA; 6 Tongji Hospital, Tongji Medical College, Huazhong University of Science and Technology, Wuhan 430030, China; wangge@hust.edu.cn; 7 School of Nursing, University of Alabama at Birmingham, Birmingham, AL 35294, USAdevance@uab.edu (D.E.V.); 8 Heersink School of Medicine, University of Alabama at Birmingham, Birmingham, AL 35233, USA; 9 School of Medicine, University of Mississippi Medical Center, Jackson, MS 39216, USA; dkparker@umc.edu; 10 School of Optometry, State Univeristy of New York, New York, NY 10036, USA; 11 School of Medicine, Emory University, Atlanta, GA 30322, USA

**Keywords:** combination antiretroviral therapy (cART), cognition, ingenuity pathway analysis (IPA), people living with HIV (PLWH)

## Abstract

**Background/Objectives:** Individual plasma protein biomarkers have been shown to correlate with cognitive performance in people with HIV (PWH). This study aimed to investigate the association between plasma proteomic signatures and attention/working memory in virologically well-controlled women with HIV (WWH). **Methods:** Seventy-seven WWH from three Women’s Interagency HIV Study (WIHS) sites completed neuropsychological (NP) testing and a blood draw. Selected protein biomarkers (200 total) were analyzed using a multiplexing method. **Results:** Random forest analysis was used to identify the top 10 biomarkers that were each positively or negatively associated with attention/working memory. Ingenuity pathway analysis (IPA) was used to facilitate data interpretation. Tumor necrosis factor receptor 1 (TNF RI), TNF RII, interleukin 1 receptor 1 (IL-1RI), and IL-6R were negatively associated with attention/working memory. **Conclusions:** Based on the IPA, two gene signaling networks were proposed for associating these plasma protein biomarkers with attention/working memory function. This novel methodology demonstrates how gene networks can be identified using blood draws in conjunction with cognitive assessment, and then used in random forest analysis, to derive value that can be put in IPA.

## 1. Introduction

Combination antiretroviral therapy (ART) can lead to successful viral suppression, which has substantially extended the lifespan of people with HIV (PWH) [1]. Many PWH experience subtle to mild cognitive impairment [2,3]. A meta-analysis of 18 neurocognitive HIV studies showed that 44.9% of PWH have HIV-associated neurocognitive disorder (HAND) [4]. Yet, there is debate about the underlying mechanisms contributing to cognitive impairment in many PWH continues. Insights on protein biomarkers as well as their related signaling pathways may help clinicians to follow the clinical course of cognitive changes in PWH and help diagnose and treat cognitive impairment.

To date, some studies have examined the association between single plasma biomarkers and cognition in PWH. Many biomarkers of immune activation and inflammation have been examined including: CXCL10 [5], interleukin (IL)-6, monocyte chemotactic protein (MCP)-1, soluble CD14 (sCD14), soluble CD163 (sCD163), and soluble TNF receptors 1 and 2 [6]. Higher peripheral MCP-1 levels, also known as (C-C motif) ligand 2 (CCL2), have been associated with cognitive impairment or decline [7,8,9,10]. From a Nigerian study, the plasma level of MCP-1 was greater among those with HAND than those without cognitive impairment [10]. Likewise, sCD14 has been suggested as a biomarker to monitor the progress of HAND as it commonly relates to cognitive impairment in PWH on ART [11]. Compared to those with unimpaired cognition, plasma sCD14 was higher in those with asymptomatic neurocognitive impairment or mild neurocognitive disorder [10], and higher levels were associated with worse global cognitive performance in PWH [12]. Similarly, sCD163 in plasma has been shown to be elevated in cognitively impaired PWH despite viral suppression [13]. Moreover, individuals with HAND have demonstrated significantly higher plasma sCD163 than those with asymptomatic cognitive impairment or normal cognition [13]. Albeit, based on the findings from an East African cohort study, sCD14 but not sCD163 was associated with cognitive performance regardless of a positive or negative HIV serum status [14]. Both sCD14 and sCD163, but not IL-6, were found to be associated with domain-specific cognitive function as well as overall performance in a group of women with HIV (WWH) (*n* = 253) with virological suppression [15]. It is noteworthy that WWH may have more prominent cognitive impairment than their male counterparts [16].

Impaired attention/working memory has been more commonly observed among PWH compared to people without HIV. For instance, in a clinical trial reported by Rarinpour et al., 30 men with HIV who used substances had poorer verbal working memory than their risk-matched seronegative controls [17]. Similarly, auditory working memory was found to have significantly more deficits in PWH when the function was compared between 41 men with HIV and 37 men without HIV who used substances [18]. In a report by Kanmogne et al., PWH (*n* = 347) had significantly lower attention/working memory scores than the seronegative controls (*n* = 395) [19].

Although single biomarkers have some predictive value for cognition, it is important to understand the disease processes underlying these biomarkers. More specifically, microbial translocation, metabolic dysregulation, heart disease, or HIV itself can cause these biomarkers to vary [20]. Identifying the underlying disease processes can not only provide further diagnostic value but also provide insights into therapeutic approaches. While many studies focus on one or a small panel of inflammatory markers, a large panel of plasma biomarkers might more accurately predict cognitive performance precisely due to the complex mechanism underlying the cognitive impairments seen in PWH [21]. For example, in a study by Aparicio et al., a blood draw and neuropsychological test battery were completed in 33 PWH [22]; plasma miRNA extraction was conducted followed by array hybridization; the top 10 miRNAs that either downregulated or upregulated cognition were identified using random forest analysis [22].

Adding additional methodological innovative to the Aparicio et al.’s study, the purpose of our study was to test the feasibility and pilot a novel technique to identify plasma proteomic signatures in relation to cognitive function (i.e., attention/working memory) in virally well-controlled WWH. Using a multiplexing method, 200 protein biomarkers were measured using plasma samples. These 200 protein biomarkers were selected from choice of convenience, which have five categories: inflammatory factors, growth factors, chemokines, receptors, and cytokines. A random forest model was used to rank the association between these protein biomarkers and attention/working memory. Random forest model can be used to process large amount of data and sort out the strong correlation between the level of biomarker(s) and cognitive function of attention/working memory. In addition, ingenuity pathway analysis (IPA) was used to determine the signaling pathway for attention/working memory function. IPA is a bioinformatics program in which researchers can upload data from microarrays, metabolomics, SNP, mi RNA, RNA-Seq gene expression, and plasma proteomic multiplexing data to identify patterns in these data that best represent networks of biological systems.

## 2. Methods

### 2.1. Participants

**Figure 1:**
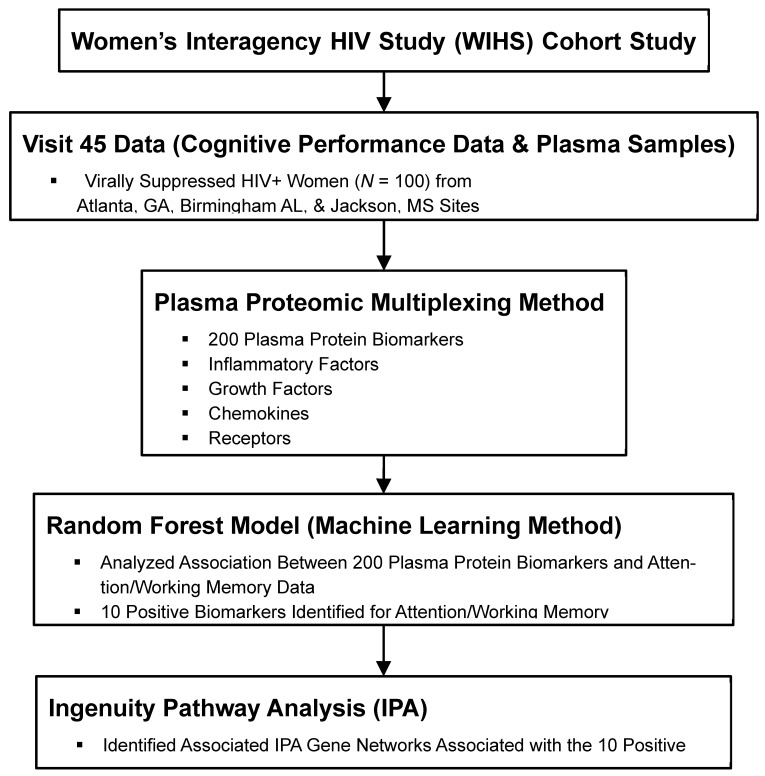
Plasma Proteomic Multiplexing—Random Forest—Ingenuity Pathway Analysis of Cognition Methodological Framework.

Ethical approval of this study was granted by the Institutional Review Board of University of Alabama at Birmingham’s in July 2021 (registration number- IRB300006874, approval date 9 July 2021). Informed consent was obtained from all subjects involved in the study. By working with the Data Analysis and Coordination Center (DACC) of the Multicenter AIDS Cohort Study (MACS)/Women’s Interagency HIV Study (WIHS) Combined Cohort Study (MWCCS), a subgroup of 100 participants was identified (participants were seen between 1 October 2016 to 31 March 2017), who were from three study sites: the University of Alabama at Birmingham (UAB), University of Mississippi Medical Center (UMMC), and Emory University (Figure 1). Selected participants had consistent viral suppression as indicated by low plasma HIV RNA as well as CD4 T cell count. Samples from participants were only included if they were virally suppressed because we wanted to know if other underlying mechanisms other than viral infection can be used to explain the increased risk of cognitive impairment commonly seen in many PWH. Cognitive data were extracted from the MWCCS database and repositories. In the database, CD4 count and plasma HIV RNA were measured concurrently with neuropsychological (NP) testing. ART history and nadir CD4 count were obtained from chart review and self-report (Table 1). Plasma HIV RNA was below the limits of detection at <20 copies/µL.

### 2.2. Measurement of Biomarkers

Fasting specimens were collected at any time of day provided the participant had nothing to eat or drink except water of the eight hours prior to phlebotomy. Further, 8 mL whole blood was collected from each participant using Mononuclear Cell Preparation Tube, which has sodium citrate as the anticoagulant. One hundred plasma samples from selected participants were retrieved from the biospecimen repository with the coordinating MWCCS DACC. These 100 participants (with their plasma samples retrieved) had achieved excellent viral suppression via ART with HIV viral RNA undetectable in blood plasma and CD4 T-cells are more than 400 per cubic millimeter of blood. Using existing panels of Quantibody arrays (QAH-CAA-4000) (RayBiotech Life, Inc., Atlanta, United States of America.), 200 plasma protein biomarkers were measured in each plasma sample (a list of them can be found in the Supplemental File). These protein biomarkers belong to five different categories: inflammatory factors, growth factors, chemokines, receptors, and cytokines.

### 2.3. Attention/Working Memory

A panel of neuropsychology experts developed the WIHS neurocognitive test battery to facilitate the diagnosis of HAND. Attention/working memory was assessed with the Letter-Number Sequencing test (outcomes = total correct on attention/working memory conditions). A higher score corresponds to better performance. Like other large-scale HIV cohorts [23,24,25] including the WIHS [24,25,26,27,28,29], sociodemographically adjusted T-scores were derived for attention/working memory. The T-scores were standardized to have a mean of 50 and a standard deviation of 10. Out of the 100 participants who had their plasma samples, only 77 had complete NP data available for analysis.

### 2.4. Statistical Analyses

**Figure 2:**
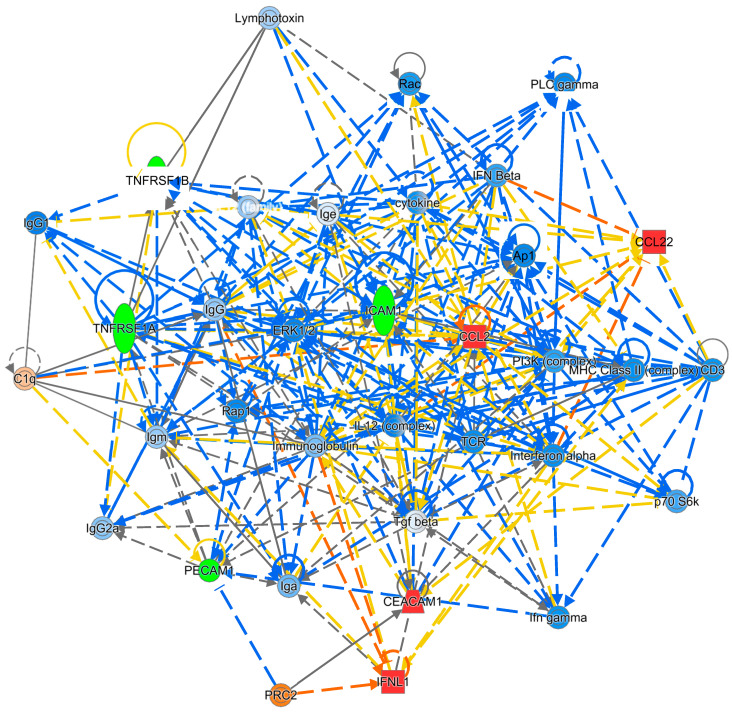
IPA Gene Network 1 for the Attention/Working Memory Function. *Note.* Green: Negatively associated biomarker; Red: Positively associated biomarker. The colors of the lines connecting genes and molecules represent the relationship between them (predicted activation or inhibition status). Blue: a predicted inhibition; Orange: a predicted activation; Yellow: the findings from our dataset are inconsistent with predicted state of the downstream molecule.

**Figure 3:**
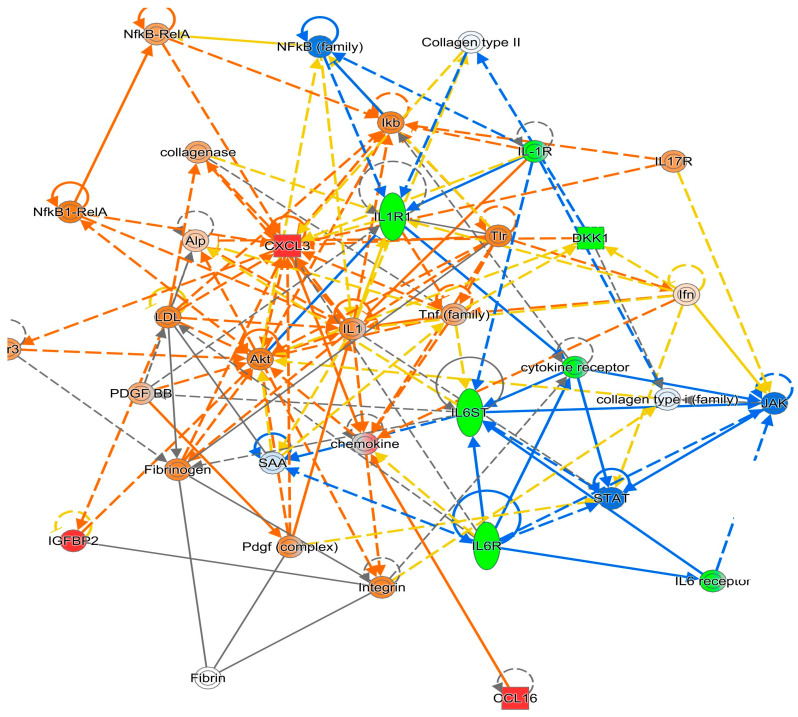
IPA Gene Network 2 for the Attention/Working Memory Function. *Note*. Green: Negatively associated biomarker; Red: Positively associated biomarker. The colors of the lines connecting genes and molecules represent the relationship between them (predicted activation or inhibition status). Blue: a predicted inhibition; Orange: a predicted activation; Yellow: the findings from our dataset are inconsistent with predicted state of the downstream molecule.

Two hundred plasma biomarkers from 77 participants were analyzed for their association with cognitive performance. To identify plasma protein biomarkers that relate to cognition, a random forest model (machine learning method) was fitted to predict scores in the domain of attention/working memory. A random forest model is a versatile machine learning tool for both classification and regression analysis. It operates by constructing a forest (array) of multiple decision trees during training and then combining their individual predictions to produce a more robust and accurate prediction. This model included all 200 biomarkers as predictors and controlled for biological sex, years of education, CD4^+^ nadir, and undetectable viral load (vs. detectable). Prior to analysis, plasma biomarker concentrations were log-transformed (log(x + 0.001)) and standardized (z-scored). The model was implemented in R using the caret package to perform hyperparameter tuning via repeated 5-fold cross-validation. The final model was built with 1000 trees (ntree = 1000), and a random seed was set (set.seed(10)) to ensure reproducibility. Variable importance was quantified using the percent increase in mean squared error (%IncMSE) upon permutation of each variable. From the fitted model, the top 10 positive and top 10 negative protein biomarkers, as ranked by their % incMSE importance scores, were identified for their association with attention/working memory. All random forest models were conducted in R, package ‘Random Forest’ version 4.6–14. These data were then transformed to mimic gene expression data to facilitate the IPA (Qiagen, Redwood City, CA, USA) [15]. For attention/working memory function, the top 10 positive correlating markers were given an expression value of 1 and a *p*-value of 0.01. The top 10 negative correlating markers were assigned an expression value of −1 and a *p*-value of 0.01. All other markers were given an expression value of 0 and a *p*-value of 1. This transformed data table was imported to the IPA as an expression dataset; actual values were not used because IPA was not designed to work with such data. Transformed data were analyzed using IPA’s core analysis with default settings. To address bias introduced by the non-random selection of markers for the expression assay, three randomization tests (similar to a bootstrapping method) were run to identify associations with the probe set itself. For each test, every protein biomarker was given a 10% chance of being significant (*p* = 0.01). For those that were significant, each had a 50% chance of having an expression value of either 1 or −1. Pathways and terms significantly associated with any of the gene lists from the three randomization runs were filtered from the analysis results. Figures were created by the IPA, which is based on a comprehensive database of known relationships from the literature; these gene networks represent the possible signaling pathways involved with the relevant protein biomarkers (Figure 2 and Figure 3).

## 3. Results

Table 1 provides the sociodemographic, behavioral, and clinical characteristics of the study sample. The participants (*n* = 77) were on average 48.0 years old (*SD* = 8.9) and reported an average education of 12.2 years (*SD* = 2.2). For race/ethnicity, 90% of the participants were Black and the remaining 10% were White. The selected participants had excellent control of their HIV infection as 97% of the participants had undetectable HIV RNA levels (less than 20 copies/mL) and the remaining 3% had detectable HIV RNA but the viral load was very low (~30 copies/mL) with the mean CD4^+^ lymphocyte count being 743 per cubic millimeters (*SD* = 333) (Table 1). The mean CD4^+^ nadir was 284.5 per cubic millimeters (median = 253). The adherence rate to ART was 94% and the average duration of ART exposure was 7.3 years (*SD* = 2.7). Seventeen percent of the participants had a prior diagnosis of acquired immune deficiency syndrome (AIDS). Thirty-eight percent were current cigarette smokers, 12% were heavy alcohol users (i.e., 5–6 drinks/day), and 27% were recent illicit substance (including marijuana) users.

**Figure 4:**
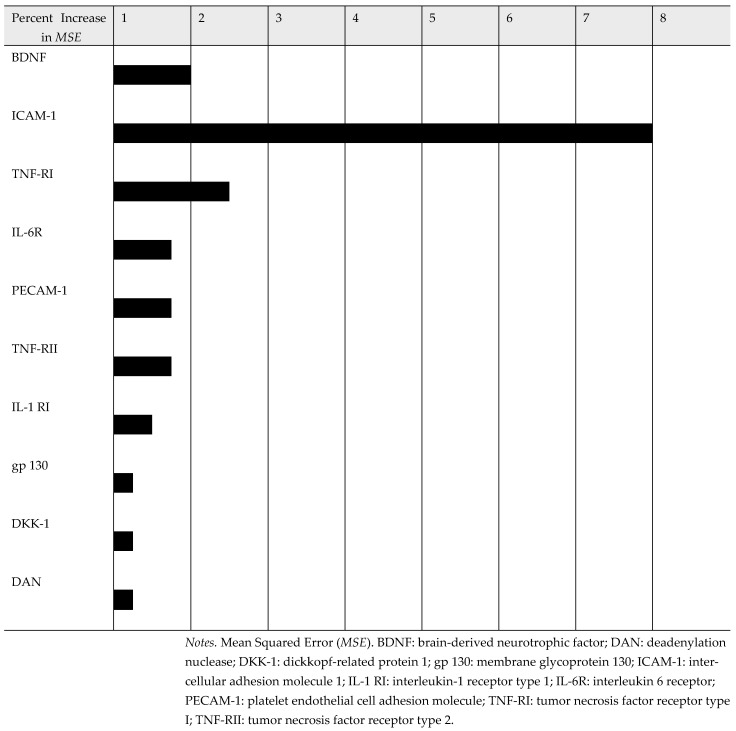
Top 10 Plasma Protein Biomarkers Correlated Negatively with Attention/Working Memory. Black bar: mean squared error fold change.

**Figure 5:**
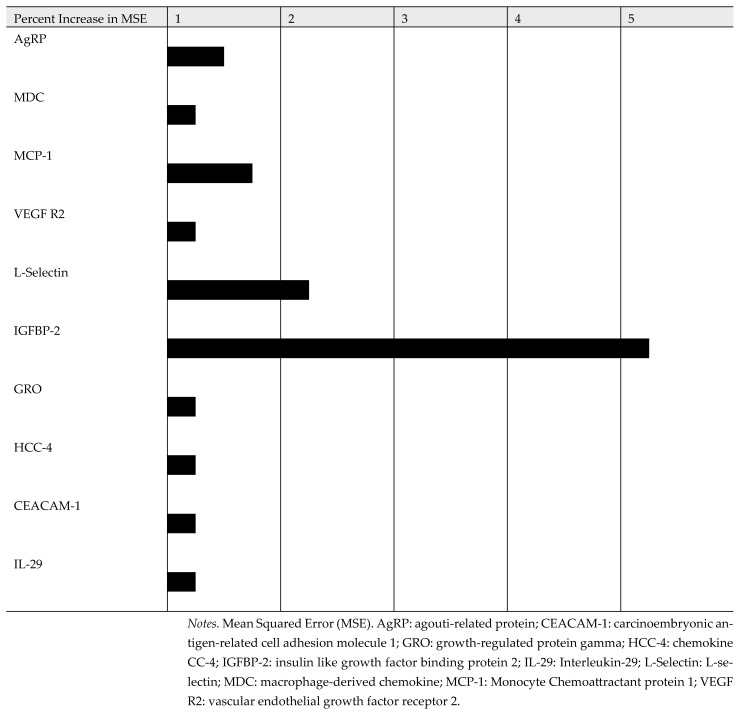
Top 10 Plasma Protein Biomarkers Correlated Positively with Attention/Working Memory. Black bar: mean squared error fold change.

Based on the random forest machine learning analysis, the top 10 positive and 10 negative protein biomarkers were identified for the attention/working memory (see Figure 4 and Figure 5). A longer line represents a stronger correlation between each specific biomarker and attention/working memory function. The top 10 plasma protein biomarkers, which were positively associated with attention/working memory function, were agouti-related protein (AgRP), macrophage-derived chemokine (MDC), monocyte chemoattractant protein 1 (MCP-1), vascular endothelial growth factor receptor 2 (VEGF R2), L-selectin, insulin like growth factor binding protein 2 (IGFBP-2), growth-regulated protein gamma (GRO), chemokine CC-4 (HCC-4), carcinoembryonic antigen-related cell adhesion molecule 1 (CEACAM-1), and interleukin-29 (IL-29). By contrast, the top 10 plasma protein biomarkers, which were negatively associated with attention/working memory function, were brain-derived neurotrophic factor (BDNF), intercellular adhesion molecule 1 (ICAM-1), tumor necrosis factor receptor type I (TNF-RI), interleukin-6 receptor subunit alpha (IL-6R), platelet endothelial cell adhesion molecule (PECAM-1), tumor necrosis factor receptor type 2 (TNF-RII), interleukin-1 receptor type 1 (IL-1 RI), membrane glycoprotein 130 (gp130), dickkopf-related protein 1 (DKK-1), and deadenylation nuclease (DAN).

Using IPA, signaling networks as well as the involved proteins were proposed based on the most relevant proteins shown in Table 2. For attention/working memory function, IL-1RI, IL-6R, tumor necrosis factor receptor 1 (TNF RI, also known as tumor necrosis factor receptor superfamily member 1A (TNFRSF1A)), and TNFR II (also known as TNFRSF1B) showed negative associations. For attention/working memory function, two possible gene networks were proposed. The possible signaling networks are shown in Figure 2 and Figure 3.

## 4. Discussion

In recent years, plasma biomarkers have attracted a lot of interest for their potential use to predict or diagnose cognitive impairment and follow cognitive trajectories in PWH. In this study, we investigated the relationship between plasma protein biomarkers and different cognitive functions in people with virally well suppressed HIV. Therefore, while these findings are meaningful to virally suppressed PWH, they should be cautiously considered for those without HIV as those who are virally suppressed may still lack immunological restoration and experience chronic immune activation and inflammation.

In our sample of WWH, TNF RI and TNF RII were negatively associated with attention/working memory function. In a different study, a negative correlation between the expression of TNF RI or TNF RII and working memory was observed in healthy subjects (*n* = 69) and patients with depression (*n* = 89) [30]. TNF RI and TNF RII belong to the tumor necrosis factor receptor superfamily, which exists on the cell membrane ubiquitously. It was reported that the plasma level of either TNF RI or TNF RII was higher in patients with mild cognitive impairments (MCI) (*n* = 137) than in age-matched controls (*n* = 30) (31). In addition, in that same study, the level of either TNF RI or TNF RII was associated with the Aβ40 in plasma [31]. In a different report, an increased serum level of TNF RI was associated with a higher risk of progression from MCI to Alzheimer’s Disease [32]. When TNF RI is activated, apoptosis is believed to be induced. By contrast, activation of TNF RII is pro-inflammatory and associated with pro-survival signaling [33].

Although no data on how either IL-1RI or IL-6R are associated with attention/working memory function has been reported, their roles are worth studying further as the IPA suggests both are involved in the signaling network for attention/working memory function.

Previous studies have indicated that advanced age and incomplete virologic suppression are major contributing factors to the development of cognitive impairments in PWH [34]. Although the participants of this current study had low or undetectable viral loads, we cannot exclude the possibility that incomplete viral load suppression created chronic inflammation.

Methodological strengths are recognized in our study. First, a medically stable cohort can minimize “noise” and obtain a better “signal” with a more homogenous group (e.g., excellent viral load control, same gender, and all Southern U.S. participation sites). By focusing on these sites, the “noise” that often comes from site location is minimized; site location has been a pervasive predictor on many cognitive tests and other study variables [35]; therefore, minimizing this “noise” should have strengthened our analyses. Therefore, for a “cleaner analysis”, a more homogeneous group was included for this analysis by focusing on WWH with viral control. Second, by measuring 200 analytes in one single plasma sample, our search for reliable protein biomarkers was much more efficient than traditional approaches [2,7,19].

Methodological limitations are also acknowledged. First, although our study was innovative for analyzing 200 proteins with the multiplexing assay method with random forest and IPA, it could present a selection bias. A selection bias could have been created when 200 protein biomarkers were chosen to be analyzed from thousands of possible candidate protein biomarkers. Second, our sample size was reduced to 77 even though protein biomarkers were analyzed from plasma samples of 100 participants due to the unavailability of cognitive data from some participants (*n* = 23). Therefore, our sample size is relatively small but decent for our analysis.

In the future, more protein biomarkers should be considered for analysis with a larger sample. For example, as many as 1000 protein biomarkers can be analyzed using one plasma sample, although the cost and benefit balance needs to be considered for casting a broader net. Instead, the findings from this study may make it possible to focus on some protein biomarkers or signaling pathways for their translational or therapeutic application value.

## 5. Conclusions

Tumor necrosis factor receptor 1 (TNF RI), TNF RII, interleukin 1 receptor 1 (IL-1RI), and IL-6R were found to be negatively associated with the attention/working memory in virally well suppressed participants with HIV. Based on the IPA, two gene signaling networks were proposed for associating these plasma protein biomarkers. This novel methodology demonstrates how gene networks can be identified using blood draws in conjunction with cognitive assessment, and then used in random forest analysis, to derive value that can be put in IPA.

## Figures and Tables

**Table 1:** Sociodemographic, Behavioral, and Clinical Characteristic of Participants (*n* = 77).

Sample Characteristics	Mean (SD) or n (%)
Age in years (Mean, SD)	48.0 (8.9)
Education in years (Mean, SD)	12.2 (2.2)
Race/ethnicity, *n* (%) Black, non-Hispanic White, non-Hispanic	69 (90) 8 (10)
Current smoking status, *n* (%)	29 (38)
Recent heavy alcohol use, *n* (%)	9 (12)
Recent marijuana use, *n* (%)	18 (23)
Recent Crack, cocaine, and/or heroin use, *n* (%)	3 (4)
Nadir CD4 count in WIHS, median (*IQR*)	284.5 (253)
Current CD4 count, median (*IQR*)	743 (333)
Undetectable HIV RNA, <20 cp/mL, *n* (%)	75 (97)
Adherence (≥95%) to cART, *n* (%)	72 (94)
ART duration in years (Mean, SD)	7.3 (2.7)
Prior AIDS diagnosis, *n* (%)	13 (17)

*Notes.* ART: Antiretroviral Therapy; IQR: Interquartile Range; *M*(*SD*): Mean (Standard Deviation).

**Table 2:** Attention/Working Memory Function, Plasma Protein Biomarkers, and Associated IPA Gene Networks.

Cognition	TNFRSF1A	TNFRSF1B	IL1R1	IL6R	Network
Attention Working Memory	**x**	**x**			**1**
			**x**	**x**	**2**

*Notes.* TNFRSF1A: tumor necrosis factor receptor superfamily member 1A; TNFSF1B; tumor necrosis factor receptor superfamily member 1B; IL1R1: interleukin 1 receptor 1; IL6R: interleukin 6 receptor.

## Data Availability

All data are available upon request by contacting the corresponding author with following the data sharing policy from the University of Alabama at Bimingham.

## Supplementary Materials

The following supporting information can be downloaded at: https://www.mdpi.com/article/10.3390/diagnostics15202649/s1.
